# A data-driven approach to optimizing waiting times in outpatient pharmacy services: Interrupted time series analysis

**DOI:** 10.1016/j.rcsop.2025.100672

**Published:** 2025-10-24

**Authors:** Hazzaa Alghamdi, Talal S. Alshihayb, Yazeed Alharbi, Mohammad Alawagi, Abdullah Aleissa, Yasser Albogami

**Affiliations:** a Pharmaceutical Care Division, King Faisal Specialist Hospital and Research Center, Riyadh, Saudi Arabia; b Department of Preventive Dental Science, College of Dentistry, King Saud bin Abdulaziz University for Health Sciences, Riyadh, Saudi Arabia; c Department of Health Policy and Health Services Research, Boston University Henry M. Goldman School of Dental Medicine, Boston, MA, USA; d Department of Clinical Pharmacy, College of Pharmacy, King Saud University, Riyadh, Saudi Arabia

**Keywords:** Outpatient pharmacy, Waiting time reduction, Operational efficiency, Data-driven intervention, Interrupted time series analysis, Queue management, Patient satisfaction

## Abstract

**Background:**

Operational efficiency in outpatient pharmacies is a critical factor in healthcare delivery, directly impacting patient satisfaction and adherence to prescribed treatments. Prolonged waiting times in pharmacies can lead to patient dissatisfaction, reduced medication adherence, and potential health risks.

**Objective:**

This study aimed to analyze the impact of a data-driven intervention on reducing patient waiting times in an outpatient pharmacy at a tertiary hospital, with a goal of ensuring that patients are served within 30 min of ticket issuance.

**Methods:**

The study utilized data from the “Qsmart” ticketing system, covering October 2022 to November 2023. A descriptive analysis was conducted to identify peak service hours and assess staffing patterns. An interrupted time series analysis (ITSA) was employed to evaluate the effectiveness of an intervention implemented between January 22 and February 26, 2023. The intervention included increased staffing during peak hours, adjustments to break schedules, and enhanced pre-peak hour preparations.

**Results:**

The descriptive analysis revealed peak service hours between 9 AM and 11 AM, with the highest number of tickets issued at 10 AM. The intervention produced a significant immediate level reduction in waiting times of 0.1540 (95 %CI: 0.0421,0.2659) but there was **no additional post-intervention slope change**, indicating that the improvement was not progressively increasing over time.

**Conclusion:**

The data-driven intervention effectively reduced waiting times in the outpatient pharmacy, with significant immediate improvements observed. This study highlights the potential of strategic operational adjustments to enhance service efficiency and patient satisfaction. Further research is needed to validate the sustainability and generalizability of these findings in other settings.

## Introduction

The operational efficiency of outpatient pharmacies plays a critical role in the overall healthcare delivery system. Patient waiting time is a key metric for assessing the quality and efficiency of pharmacy services.^1^ In an era where patient satisfaction is increasingly prioritized alongside clinical outcomes, the ability of a pharmacy to dispense medications promptly is not just a measure of operational performance but also a fundamental aspect of patient care.^2^ Delays in medication delivery can lead to patient distress, reduced adherence to prescribed regimens, and in some cases, direct health risks, especially for individuals with urgent pharmaceutical needs.^3^ At the outpatient pharmacy, baseline median waiting times exceeded 25 min, with more than 40 % of patients experiencing waits longer than 30 min during peak hours.

The importance of minimizing wait times in outpatient pharmacies cannot be overstated, given the clear imperative to enhance patient satisfaction and ensure timely access to medications.^4^ Despite this, outpatient pharmacies frequently encounter a range of operational challenges that impede their ability to meet acceptable service benchmarks.^5^^,^^6^ These challenges include fluctuating patient volumes, uneven staff distributions, and systemic inefficiencies that compound the complexities of pharmacy management.^6^^,^^7^ Such operational hurdles highlight a significant opportunity for improvement in service protocols and processes, underscoring the need for strategic initiatives aimed at optimizing operational capacity and service demand. While these issues are well-documented, there is a lack of research that uses a data-driven approach to quantify and address these inefficiencies.

To address these challenges, the pharmacy implemented a bundled intervention that included increasing pharmacist coverage during peak hours, rescheduling staff breaks, and enhancing pre-peak preparation activities. These components were selected based on operational bottlenecks identified in preliminary internal audits, which showed understaffing during peaks and inefficiencies related to simultaneous staff breaks.

Previous pharmacy and outpatient clinic improvement efforts have focused on workflow redesign, automation, or Lean Six Sigma methodologies, often demonstrating reductions in waiting times by 20–50 %. However, these approaches can require substantial financial or infrastructural investment. In contrast, the current approach leverages routine service data to identify and implement low-cost operational adjustments, representing a practical and scalable model.

This study had two primary objectives. The first was to apply a robust, data-driven approach to analyze patient flow and staff performance data in order to identify operational bottlenecks and potential areas for improvement. The second objective was to evaluate the effectiveness of a bundled operational intervention in enhancing pharmacy service efficiency, as reflected by reductions in patient waiting time and adherence to the institutional key performance indicator (KPI) of serving patients within 30 min.

## Methods

### Data source and variables

Data were obtained from the ‘Qsmart’ ticketing system of the outpatient pharmacy at King Faisal Specialist Hospital and Research Centre, Riyadh, Saudi Arabia, covering the period from October 1, 2022 to November 30, 2023. The dataset includes timestamps for ticket issuance and ticket calling, as well as deidentified pharmacist IDs. Prior to analysis, data were subjected to cleaning and preprocessing to ensure accuracy and completeness.

### Study design

To address the first objective, a descriptive analysis was conducted on the three-month pre-intervention dataset (October 1, 2022 to December 31, 2022). Key metrics such as the time and day of ticket issuance, waiting time (time between ticket issuance and calling), and the number of pharmacists serving concurrently were examined to identify patterns and peak service times.

For the second objective, the study employed an interrupted time series design to evaluate the impact of the intervention, which was implemented between January 22 and **February 26**, 2023. The intervention comprised several actions aimed at optimizing service efficiency. Table 1 summarizes the intervention components, their implementation dates, and whether they were applied concurrently or sequentially.

**Table 1:** Intervention components summary.

Component	Description	Implementation Period	Concurrent/ Sequential	Expected Effect
Full staffing of 13 windows	Reassigned pharmacists from non-critical duties to ensure full coverage	Jan 22 – Feb 26, 2023	Concurrent	Increase throughput during peak hours
Extra 3–4 staff on standby	Covered breaks and supported peaks	Jan 22 – Feb 26, 2023	Concurrent	Maintain service continuity
Off-peak coverage	Maintained 9–10 windows operational during off-peak	Jan 22 – Feb 26, 2023	Concurrent	Reduce bottlenecks after peaks
Pre-peak preparations	Prepackaging meds, advance stocking	Jan 22 – Feb 26, 2023	Concurrent	Shorten dispensing phase
Rescheduled breaks	Strategic rotations during off-peak	Jan 22 – Feb 26, 2023	Concurrent	Prevent coverage gaps
Daily huddles	3× per week performance review & corrective action	Jan 22 – Feb 26, 2023	Concurrent	Continuous improvement
Dashboard monitoring	Managerial tool for real-time adjustments	Jan 22 – Feb 26, 2023	Concurrent	Rapid response to surges

### Outcome measures

The **primary outcome** was the daily median waiting time (minutes) from ticket issuance to ticket calling. The **secondary outcome** was the daily proportion of tickets served within 30 min (number served ≤30 min divided by total daily tickets), reflecting the institutional KPI.

### Statistical analysis

For the descriptive analysis, means, medians, and standard deviations were calculated for waiting times. Frequency distributions were generated to identify peak service hours based on the time and day of ticket issuance. The number of pharmacists serving concurrently during different time slots was analyzed to assess staffing patterns in relation to service demand.

To evaluate the impact of the intervention, an interrupted time series analysis^8^ was performed on the daily proportion of tickets called within 30 min of issuance. A segmented regression model was fitted to the time series data, with the intervention period as the point of interruption. This model estimated changes in the level and trend of the outcome variable before and after the intervention. Based on the data pattern and knowledge of the intervention, the exposure–outcome function was assumed to be linear. An immediate change in the outcome level was assumed to occur right after the intervention, along with a change in the trend (slope) of the outcome over time following the intervention. Model assumptions, including stationarity and independence of errors, were checked using diagnostic plots such as residual plots and autocorrelation function plots.

Sensitivity analyses were conducted to assess the robustness of the findings. These included testing alternative model specifications (level-only, slope-only, and combined level-and-slope change models), examining different functional forms for the outcome–exposure relationship (linear, polynomial, and spline models), and repeating the analysis using an alternative intervention window (February 22 to March 23, 2023) to evaluate temporal stability. These analyses aimed to confirm the consistency of the estimated intervention effects under different modeling assumptions.

All statistical analyses were performed using R software, with the following packages: *dplyr*^9^ for data manipulation, *ggplot2*^10^ for data visualization, *lubridate*^11^ for handling date and time data, stats for statistical modeling, *readxl*^12^ for reading Excel files, *tseries*^13^ and forecast for time series analysis, and *lmtest*^14^ for testing linear models. Results were presented in graphs to illustrate the observed patterns and the impact of the intervention on pharmacy service efficiency.

## Results

### Descriptive statistics

**Fig. 1:**
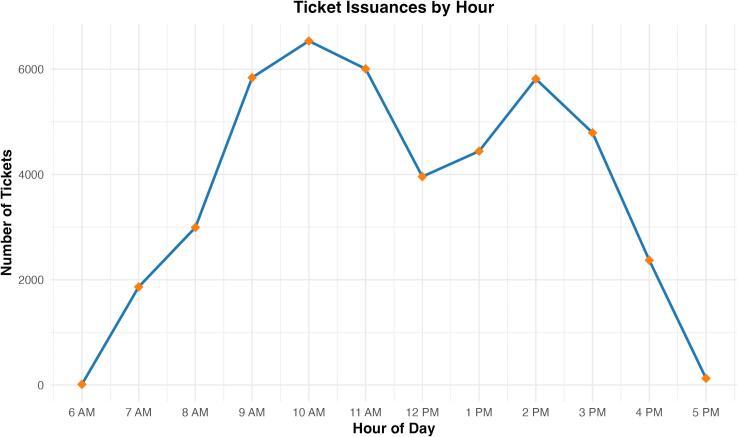
Ticket issuances by hour.

**Fig. 2:**
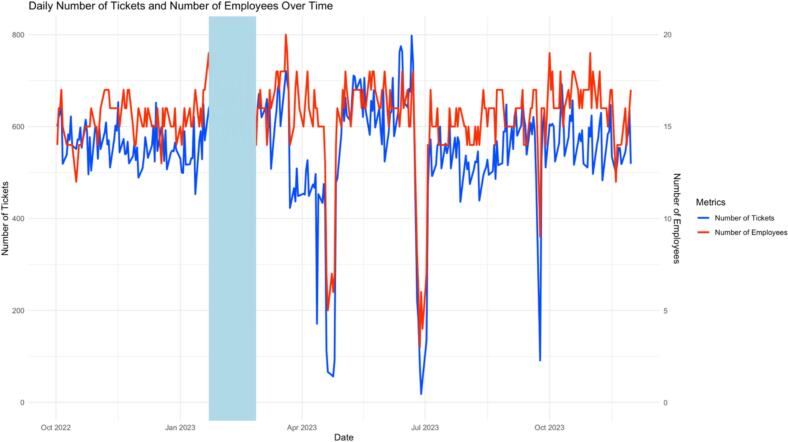
Daily number of tickets and number of employees over time.

The descriptive analysis included 44,754 tickets issued between October 1, 2022 and November 30, 2023. The primary outcome, daily median waiting time (minutes), was 24 min (IQR 16–33; mean = 25.9 min), ranging from less than 1 min to 97 min (Supplementary Table S1). The distribution of ticket issuances by hour indicated that the peak service hours were between 9 AM and 11 AM, with the highest number of tickets issued at 10 AM (Fig. 1). A notable decrease in ticket issuances was observed at 12 PM, with a slight spike occurred between 1 PM and 3 PM. The number of pharmacists serving concurrently varied throughout the day, with a minimum of 4 pharmacists at 6 AM and a maximum of 22 pharmacists between 10 AM and 1 PM. Fig. 2 presents the daily counts of tickets issued and the number of employees over time. The figure depicts fluctuations in both metrics throughout the study period.

**Fig. 3:**
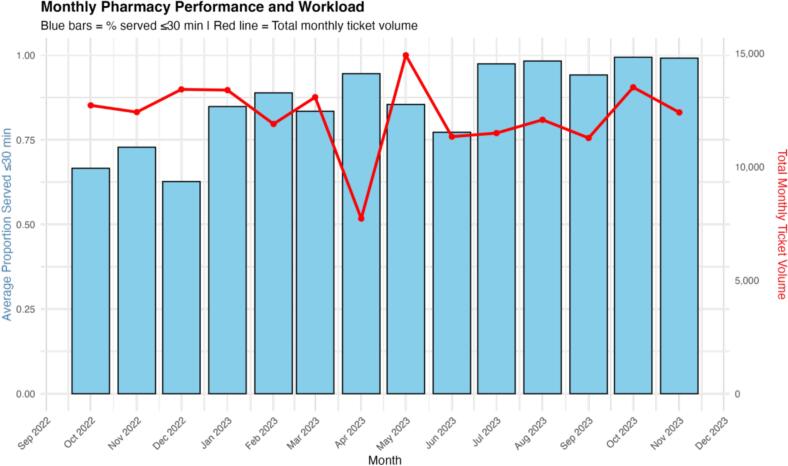
Average proportion of patients served within 30 minutes per month.

The secondary outcome, daily proportion of tickets served within 30 min (institutional KPI), showed progressive improvement throughout the study period. The average monthly proportion increased from below 75 % in early months to consistently reaching 100 % by mid-2023, indicating sustained efficiency gains in pharmacy service delivery (Fig. 3). As shown in Fig. 3, the performance trend is presented alongside the corresponding monthly prescription volume, illustrating that improvements were maintained despite fluctuations in workload. A chi-square test comparing the proportion of tickets served within 30 min before and after the intervention also showed a significant improvement (*p* < 0.001).

Waiting times varied by time of day and service type, with longer delays observed during peak hours and shorter times in urgent care services (Table 2).

**Table 2:** Waiting times by time of day and service type.

Category	≤30 min (mean, min–max)	>30 min (mean, min–max)	% ≤ 30 min
**By Time of Day**			
6–7 AM	15.2 (0.5–28)	38.7 (31–62)	83.4 %
10 AM (peak)	22.5 (5–30)	44.9 (31–97)	57.8 %
1–3 PM	20.1 (4–30)	41.2 (31–86)	∼65 %
**By Service Type**			
Fast Track	18.2 (0.5–30)	33.9 (31–72)	High
Regular Track	19.4 (0.5–30)	44.2 (31–97)	Moderate
Urgent Care Track	7.1 (0.2–20)	–	Nearly all ≤30 min

**Fig. 4:**
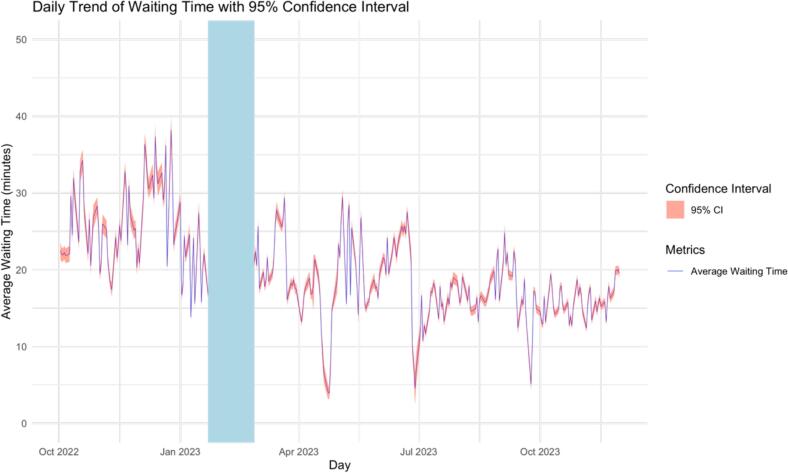
Daily trend of waiting time with 95 % confidence interval.

Fig. 4 shows the trend in patient waiting times from October 2022 to November 2023. Before the intervention, waiting times often exceeding 30 min. After implementing the intervention, there is a noticeable decrease in both average waiting times and variability, suggesting improved pharmacy service efficiency.

### Interrupted time series

The intervention increased the proportion of patients served within 30 min by approximately 0.1468 (95 %CI: 0.0869,0.2067) immediately on the first day of intervention (January 22, 2023). However, the daily trend of the proportion of patients served within 30 min did not indicate a noticeable change before or after the intervention.

An extended model incorporating the number of employees, the number of tickets issued, and the peak hour of operation each day was also analyzed. While adjusting for these variables, the intervention increased the proportion of patients served within 30 min by approximately 0.1547 immediately on the first day of intervention. However, the daily trend of the proportion of patients served within 30 min did not significantly change before or after the intervention.

An ARIMA model with regression variables for intervention and post-intervention time, in addition to the number of employees, the number of tickets issued, and the peak hour of operation each day was also fitted to the daily proportion of patients with a waiting time of less than 30 min. The model indicated that the intervention had a positive effect on the outcome, increasing the proportion of patients served within 30 min by approximately 0.1540 (95 %CI: 0.0421,0.2659).

### Sensitivity analyses

Various models were tested to assess the impact of the intervention, including models with only a level change, both level and slope changes, and a slope-only change. The results consistently showed a positive effect of the intervention on the proportion of patients with shorter waiting times.

Outcome-exposure functions were also explored using linear, polynomial, and spline models. The spline model provided the best fit based on the Akaike Information Criterion (AIC) and the Bayesian Information Criterion (BIC). However, the results were consistent with the original results.

The analysis was repeated with a different intervention period (February 22, 2023, to March 23, 2023). The segmented regression analysis again showed an increase in the proportion of patients with a waiting time of less than 30 min following the intervention (Estimate = 0.1527, 95 %CI: 0.0597,0.2457).

## Discussion

This study found that a bundled operational intervention led to a significant immediate increase in the proportion of patients served within 30 min, with no evidence of a sustained slope change. These effects were robust across multiple sensitivity analyses and consistent under peak patient load conditions. The absence of a progressive slope change may reflect a ceiling effect, as efficiency gains were realized quickly but could not continue to accumulate without additional systemic redesign.

Similar immediate impacts have been documented in other healthcare settings where a redesign of the physical space and workflow significantly reduced average waiting times from over one hour to 30 min, leading to improved patient satisfaction. While physical redesign and automation can substantially reduce waiting times, they often involve complex, time-consuming changes that are difficult to implement in many settings. In contrast, the intervention implemented in this study demonstrates that data-driven scheduling and staffing adjustments can achieve comparable immediate benefits through practical, easily deployable measures. Redesigning pharmacy layouts and workflows has also been shown to improve service efficiency and patient satisfaction. For example, a hospital pharmacy that adopted an open layout, implemented automated dispensing, and optimized workflow processes reduced average waiting times from over one hour to approximately 30 min, with a corresponding increase in patient satisfaction.^15^ Similar improvements have been documented in other healthcare settings following evidence-based process redesigns, highlighting that both structural and procedural enhancements can yield measurable efficiency gains when strategically implemented.^16^ Although patient satisfaction is likely to be affected by reduced wait times, this outcome was not directly measured in the present study. Future work should incorporate patient-reported outcomes.

The use of data-driven approaches to streamline workflows and optimize staff efficiency in healthcare settings has become increasingly prevalent. For instance, in an outpatient ophthalmology clinic, Lean Six Sigma techniques were employed to analyze patient flow and identify bottlenecks using data from time-motion studies. The data revealed that over 70 % of patient time in the clinic was spent waiting, leading to targeted interventions that included revising appointment schedules and optimizing staff assignments. These changes significantly reduced patient wait times and improved the overall efficiency of clinic operations.^17^ Moreover, a systematic review underscores the significant impact of Lean and Six Sigma (LSS) methodologies in this context. The study revealed that by utilizing data-driven tools such as the Define-Measure-Analyze-Improve-Control (DMAIC) process, hospital pharmacies achieved a 26 % reduction in medication turnaround times. This improvement was largely attributed to the precise identification and elimination of bottlenecks, informed by continuous data monitoring and analysis. Furthermore, the integration of data analytics not only streamlined pharmacy workflows but also contributed to a 15 % enhancement in overall process efficiency, demonstrating the critical role of data in driving operational excellence and improving patient satisfaction.^18^ These findings, consistent with the approach used in this study, highlight how leveraging data-driven insights can yield substantial improvements in healthcare delivery—particularly in reducing waiting times and optimizing resource utilization within hospital pharmacies. Integrating real-time dashboards and automated alerts to detect rising wait times could further help sustain these improvements.

Future work in the integration of data-driven methodologies within hospital pharmacy operations holds substantial promise for enhancing efficiency, patient outcomes, and overall healthcare quality. As healthcare systems increasingly rely on data science, the use of advanced analytics, artificial intelligence (AI), and machine learning is becoming pivotal in optimizing workflows, predicting patient needs, and managing resources more effectively. These technologies allow for the development of predictive models that can anticipate medication shortages, optimize staffing levels, and streamline inventory management, ultimately reducing waiting times and improving patient satisfaction.^19^, ^20^, ^21^, ^22^, ^23^

In the future, the application of data-driven insights to optimize healthcare workflows holds immense potential for revolutionizing patient care. By leveraging AI-powered tools, healthcare providers can predict patient demand, streamline scheduling, and manage patient flow with unprecedented precision. For example, AI algorithms can identify potential bottlenecks before they occur, allowing for proactive adjustments that enhance the efficiency of patient transitions across care settings. Real-time data analysis by AI systems will enable faster, more informed decision-making, reducing errors and improving the overall quality of care. These advancements will be particularly impactful in low-resource environments, where the ability to maintain high standards of care is crucial. AI's integration into healthcare workflows will drive significant improvements in patient outcomes and operational efficiency, marking a transformative shift in how healthcare is delivered.^21^, ^22^, ^23^

Data-driven approaches in outpatient pharmacies offer substantial economic benefits while significantly improving patient health outcomes. By utilizing predictive analytics and real-time data, pharmacies can optimize resource allocation, leading to reduced operational costs. For example, accurately forecasting patient demand allows pharmacies to manage inventory more efficiently, minimizing waste and reducing expenses associated with overstocking or understocking medications. This economic efficiency translates into cost savings, which can be reinvested into improving patient services and care quality.^24^ Moreover, data-driven strategies enhance patient health outcomes by enabling personalized care plans and more effective treatment management. By analyzing patient data, pharmacies can identify those at higher risk of complications or non-adherence to medication regimens, allowing for timely interventions that prevent costly hospital readmissions and improve overall patient health. This proactive approach not only reduces healthcare costs associated with adverse events but also promotes better long-term health outcomes, contributing to a more sustainable healthcare system.^20^^,^^25^

This study's strengths lie in its robust data-driven methodology, which leverages real-time data from the “Qsmart” ticketing system to accurately analyze patient flow and staff performance. The use of interrupted time series analysis adds statistical rigor, allowing for a detailed assessment of the intervention's effectiveness while accounting for underlying trends. The inclusion of sensitivity analyses further bolsters the study's credibility, demonstrating the robustness of the findings across different scenarios and reinforcing the reliability of the observed improvements. However, the study also has limitations that warrant consideration. Conducted within a single outpatient pharmacy in a tertiary hospital, the findings may not be generalizable to other settings, particularly those with different operational challenges or patient demographics. The relatively short post-intervention follow-up period raises concerns about the sustainability of the observed improvements over time. Potentially time-varying unobserved confounding may arise if unmeasured factors differ between the pre- and post-intervention periods and influence waiting times through pathways not captured by the observed number of tickets. For example, variations in patient attendance patterns or clinic visit behaviors across periods could affect waiting times independently of the recorded ticket volumes. The assumption of a linear exposure-outcome relationship may oversimplify complex interactions within the data although it was assessed in the sensitivity analyses. Additionally, the focus on quantitative analysis may overlook qualitative insights, such as patient and staff experiences, that could provide a more comprehensive understanding of the intervention's impact. Moreover, any concurrent changes in the hospital (in addition to the policy implemented) could have affected the impact of the policy studied.

In conclusion, the intervention produced an immediate and measurable reduction in waiting times. Replicating this approach requires only routine ticketing data and flexible staffing practices, making it a scalable and cost-effective option. Sustaining gains will require real-time monitoring and rapid response to emerging bottlenecks. Future studies should evaluate long-term sustainability and patient-reported outcomes.

## Data avilability

The datasets generated and/or analyzed during the current study are available from the corresponding author on reasonable request. Data will be shared in compliance with applicable privacy laws, ethics regulations, and institutional guidelines.

## CRediT authorship contribution statement

**Hazzaa Alghamdi:** Writing – review & editing, Project administration, Data curation, Conceptualization. **Talal S. Alshihayb:** Writing – original draft, Methodology, Formal analysis. **Yazeed Alharbi:** Writing – review & editing, Resources, Conceptualization. **Mohammad Alawagi:** Writing – review & editing, Resources, Project administration, Conceptualization. **Abdullah Aleissa:** Writing – review & editing, Resources, Project administration, Conceptualization. **Yasser Albogami:** Writing – original draft, Visualization, Project administration, Methodology, Funding acquisition, Formal analysis, Conceptualization.

## Ethics approval and consent to participate

The study was approved by the ethics committee of King Faisal Specialist Hospital & Research Centre. Due to the retrospective nature of the study and the use of de-identified data, the requirement for informed consent was waived. We ensured that the study was conducted in strict accordance with the principles of the Declaration of Helsinki.

## Funding

None.

## Declaration of competing interest

The authors declare that they have no known competing financial interests or personal relationships that could have appeared to influence the work reported in this paper.

## Appendix A Supplementary data

Supplementary material Image 1
