# Supplementary material for: A data-driven approach to optimizing waiting times in outpatient pharmacy services: Interrupted time series analysis
Source: Explor Res Clin Soc Pharm. 2025 Oct 24;20:100672. doi: 10.1016/j.rcsop.2025.100672 (PMC12639288; doi:10.1016/j.rcsop.2025.100672)
Supplement: Supplementary file 1 — Supplementary material [file mmc1.docx]

**Supplementary Table S1.** Daily Waiting Time Distribution

| **Statistic** | **Value (minutes)** |
| --- | --- |
| Mean | 25.9 |
| Median | 24 |
| Standard Deviation | 14.4 |
| Minimum | 0.12 |
| 1st Quartile (Q1) | 16 |
| 3rd Quartile (Q3) | 33.35 |
| Maximum | 97.1 |
